# P2X Receptors Inhibit NaCl Absorption in mTAL Independently of Nitric Oxide

**DOI:** 10.3389/fphys.2017.00018

**Published:** 2017-01-24

**Authors:** Samuel L. Svendsen, Søren Isidor, Helle A. Praetorius, Jens Leipziger

**Affiliations:** Department of Biomedicine, Physiology, Aarhus UniversityAarhus, Denmark

**Keywords:** purinergic, renal transport, loop of Henle, P2 receptors, P2X_4_

## Abstract

Activation of basolateral P2X receptors markedly reduces NaCl absorption in mouse medullary thick ascending limb (mTAL). Here we tested the role of nitric oxide (NO) in the ATP-mediated (P2X) transport inhibition. We used isolated, perfused mTALs from mice to electrically measure NaCl absorption. By microelectrodes we determined the transepithelial voltage (V_te_) and transepithelial resistance (R_te_). Via these two parameters, we calculated the equivalent short circuit current, I'_sc_ as a measure of the transepithelial Na^+^ absorption. Basolateral ATP (100 μM) acutely induced reversible inhibition of Na^+^ absorption (24 ± 4%, *n* = 10). Addition of L-arginine (100 μM) had no apparent effect on the ATP-induced transport inhibition. Acute reduction of extracellular [Ca^2+^] to either 100 nM or 0 nM by addition of EGTA had no effect on the ATP-induced transport inhibition. In the presence of the NO synthase (NOS) inhibitor L-NAME (100 μM) and/or ODQ to inhibit the guanylyl cyclase, the ATP effect remained unaffected. Increasing the concentration and incubation time for L-NAME (1 mM) still did not reveal any effect on the ATP-mediated transport inhibition. Acute addition of the NO donors SNAP (100 μM) and Spermine NONOate (10 μM) did not alter tubular transport. High concentrations of L-NAME (1 mM) in itself, however, reduced the transepithelial transport significantly. Thus, we find no evidence for nitric oxide (NO) as second messenger for P2X receptor-dependent transport inhibition in mTAL. Moreover, Ca^2+^ signaling appears not involved in the ATP-mediated effect. It remains undefined how P2X receptors trigger the marked reduction of transport in the TAL.

## Introduction

Extracellular nucleotides have widespread effects on the entire renal tubular transport system and mediate their effects via luminal and basolateral purinergic P2 receptors (Leipziger, 2003; Praetorius and Leipziger, 2010). Commonly, P2 receptor stimulation leads to inhibition of renal ion and water transport (Rieg et al., 2007; Kishore et al., 2009; Wildman et al., 2009; Praetorius and Leipziger, 2010). Nucleotide-mediated transport inhibition has been documented e.g., in the proximal tubule for ${\text{HCO}}_{3}^{-}$ absorption or in the collecting duct for AQP2- or ENaC-meditated water or Na^+^ transport (Kishore et al., 1995; Lehrmann et al., 2002; Bailey, 2004; Pochynyuk et al., 2008). Luminal and basolateral nucleotides are present in sufficient concentrations that generates a local paracrine “purinergic tone” that imposes transport inhibition and thus, a paracrine diuretic effect (Praetorius and Leipziger, 2010). The thick ascending limb of the loop of Henle shows functional expression of luminal and basolateral P2Y_2_ receptors and basolateral P2X receptors (Jensen et al., 2007; Marques et al., 2012). Direct transport studies in mice demonstrate that P2Y_2_ receptors are not implicated in either acute or chronic regulation of ion absorption in this segment (Marques et al., 2012, 2013). Acute application of basolateral UTP showed neither effects on transepithelial electrical transport parameters (Marques et al., 2012) nor on O_2_ consumption (Silva and Garvin, 2009) and the genetic absence of P2Y_2_ receptors does not substantially affect baseline or AVP-stimulated transport properties in TAL as measured with electrophysiological means (Marques et al., 2013). In contrast, ATP applied basolaterally, caused substantial (~25%) and reversible inhibition of Na^+^ and Cl^−^ absorption. In suspensions of rat mTALs, ATP reduced O_2_ consumption, which similar to the transepithelial voltage reductions is an indirect readout for ion transport inhibition (Silva and Garvin, 2009). In both studies, the ATP effect was shown to be mediated by P2X receptors, which via knock-out mice was demonstrated to include P2X_4_ receptors (Marques et al., 2012). Noteworthy, comprehensive transcriptome analysis shows the P2X_4_ receptor to be the only P2X receptor readily detectable in several rat tubular segments including the TAL (Lee et al., 2015). A recent study also reports an inhibitory effect of the P2X_4_ receptor on the TRPM6 Mg^2+^ channel expressed in distal convoluted tubule further supporting the widespread inhibitory effects of extracellular ATP on solute transport (de Baaij et al., 2014). It is an interesting aspect of renal tubular physiology that P2X receptors, which essentially are non-selective cation channels, can regulate tubular transport but it remains unknown how P2X receptors transduce their action. In suspension of rat TAL, ATP, UTP and β,γ-Me-ATP all increase DAF-2 fluorescence suggesting cytosolic production of NO in response to these substances (Silva et al., 2006). The authors proposed that P2X receptors stimulate the formation of NO leading to the production of cGMP and subsequent cGMP-dependent kinase mediated inhibition of the apical NKCC2 transporter. This hypothesis was based on data, which showed that the ATP-induced reduction of O_2_ consumption was absent in the presence of 3 mM L-NAME (Silva and Garvin, 2009). To further characterize the signal transduction pathway of P2X receptors in mTAL that markedly inhibits Na^+^ and Cl^−^ absorption, we adapted the idea of NO as messenger in this process and tested this hypothesis by measuring the transepithelial transport in isolated perfused mouse mTAL. We do, however, find no evidence for NO signaling in the P2X receptor-dependent transport inhibition in mouse mTAL.

## Materials and methods

### Animals

All procedures involving mice and housing of the mice were carried out according to Danish legislation (Executive order no. 12 7th of January 2016) and the EU Directive 2010/63/ on the protection of animals used for scientific purposes. Experiments were performed using 4–7 week old mice of either sex. Animals had free access to standard rodent diet and tap water. In this study the mice were of C57/B6 background were bred in house or purchased from Taconic, Silkeborg, Denmark or Janvier Laboratories, Le Genest-Saint-Isle, France.

### Tubule perfusion and measurement of ion transport in mTAL

Mice were killed by cervical dislocation complying with Danish legislation (Executive order no. 12 7th of January 2016) and the EU Directive 2010/63/ on the protection of animals used for scientific purposes. Both kidneys were removed and placed in ice-cold control solution (see below) before slicing as previously described (Wright et al., 1990). The slices were transferred into a dissection chamber cooled to 4°C. Medullary TALs were isolated in control solution (see below) from the inner stripe of the outer medulla (ISOM) by fine forceps. Kidney tubules were transferred to a perfusion chamber on an inverted microscope and perfused by a system of concentric glass pipettes (Greger and Hampel, 1981). The tubule was placed directly on the glass bottom of the experimental chamber and perfused from one side leaving the other end open. A holding pipette was often used to stabilize the preparation. The measurement of ion transport in isolated perfused tubules was carried out as described before using a double-barreled perfusion pipette (Lehrmann et al., 2002). Shortly, the transepithelial voltage difference (V_te_) was measured via one barrel between the lumen and a reference electrode placed in the bath. Via a silver wire placed into the other barrel a small rectangular current pulse of 13.0 nA (I_0_) was injected in the tubular lumen and measured as a voltage deflection (ΔV_0_) at the perfusion side. The following cable equation was then used to determine the resistance of the tissue (R_te_ in Ω·cm^2^):

$${\text{R}}_{\text{te}}\;=\;2\cdot \sqrt{\Pi \cdot \;\rho \cdot \;\lambda^{3}\cdot \frac{{\Delta \text{V}}_{0}}{{\text{I}}_{0}}\cdot \text{ tanh}\frac{\text{L}}{\lambda }}$$

The length and the inner radius *r* (μm) of the tubule (L) were measured by a size calibrated transmission image. Previously obtained resistivity values ρ (Ω^*^cm) of the perfusate were used. We only used long tubules (>400 μm) and the length constant λ was determined by the following equation (Greger, 1981):

$$\lambda \;=\;\frac{\Delta {\text{V}}_{0}\cdot \;\Pi \cdot \;{\text{r}}^{2}}{{\text{I}}_{0}\cdot \rho }$$

Applying Ohm's law, the equivalent short circuit current (I'_sc_ in μA/cm^2^) was then calculated

$$\text{I}\prime_{\text{sc}}\;=\;\;\frac{{\text{V}}_{\text{te}}}{{\text{R}}_{\text{te}}}[\mu \text{A}/\text{c}{\text{m}}^{2}]$$

and is a quantitative approach to assess electrogenic ion transport in renal tubules including the mTAL (Greger, 1985).

### Measurement of intracellular Ca^2+^ concentration in perfused mTAL

Perfused TALs were stabilized on the bath bottom with a holding pipette. The perfusion chamber was mounted on an inverted fluorescence microscope (Axiovert 100 TV, Zeiss, Germany). The setup comprises an inverted microscope with a 63x C-Apochromat 1.2NA water objective and a digital CCD camera (Spot Pursuit 1.4 monochrome, Diagnostic Instruments, Sterling Heights, USA). Images were acquired and data analyzed with standard software (VisiView, Visitron, Puchheim, Germany). Intracellular Ca^2+^ concentration ([Ca^2+^]_i_) was measured with the single wavelength fluorescent dye fluo-4 AM (Invitrogen, USA). Tubules were incubated with 5 μM basolateral fluo-4-AM in control solution for 30 min at room temperature during continuous luminal perfusion, followed by a 10 min basolateral washout period at 37°C. Fluo-4 fluorescence was measured every 3 s using 480 nm excitation for 300 ms. A 500 nm beam split and a 520–560 nm band pass filter were used to collect the emitted light using binning factor 3. Experimental manipulations were carried out after a stable fluorescence signal was achieved. Fluorescence of the entire tubule was recorded and used for data analysis after background light subtraction. ATP-induced changes of [Ca^2+^]_i_ were expressed normalized as F/F_0_.

#### Solutions and chemicals

Experiments were performed at 37°C with the following control solution: (in mM) 145 NaCl, 1 MgCl_2_, 1.3 Ca-gluconate, 5 D-glucose, 0.4 KH_2_PO_4_, 1.6 K_2_HPO_4_, 5 HEPES. This solution was also used for tubule dissection. The solution with reduced (100 nM) Ca^2+^ contained: (in mM) 145 NaCl, 1 MgCl_2_, 0.726 Ca-gluconate, 1 EGTA, 5 D-glucose, 0.4 KH_2_PO_4_, 1.6 K_2_HPO_4_, 5 HEPES. The solution free of Ca^2+^ contained: (in mM) 145 NaCl, 1 MgCl_2_, 5 EGTA, 5 D-glucose, 0.4 KH_2_PO_4_, 1.6 K_2_HPO_4_, 5 HEPES. Solutions were titrated with NaOH or HCl to pH 7.4 (37°C).

All chemicals were obtained from Sigma-Aldrich Denmark (Vallensbaek, Denmark), Tocris Bioscience (Bristol, UK) and Merck (Darmstadt, Germany). Agonist solutions were prepared directly before the experiment. *Abbreviations* used: L-NAME: N^5^-[imino(nitroamino)methyl]-L-ornithine, methyl ester, monohydrochloride; ODQ: 1H-(Alderton et al., 2001; Bailey, 2004; de Baaij et al., 2014) oxadiazolo[4,3-a]quinoxalin-1-one; SNAP: S-Nitroso-N-Acetyl-D,L-Penicillamine; oATP: Adenosine 5'-triphosphate, periodate oxidized sodium salt; ATP: Adenosine 5'triphosphate disodium salt hydrate.

#### Statistics

Data are shown as mean ± standard error of the mean (SEM). For experimental series *n* reflects the number of tubules used. On average 2 tubules were used from each mouse. Normal distribution was confirmed by the Kolmogorov–Smirnov test. For non-normal distributed series the Mann–Whitney test was used to compare mean values. Otherwise, paired *t*-tests were used to compare mean values within one experimental series. A *p*-value of <0.05 was accepted to indicate statistical significant differences.

## Results

### Basolateral ATP inhibits NaCl absorption in mouse mTAL

**Figure 1:**
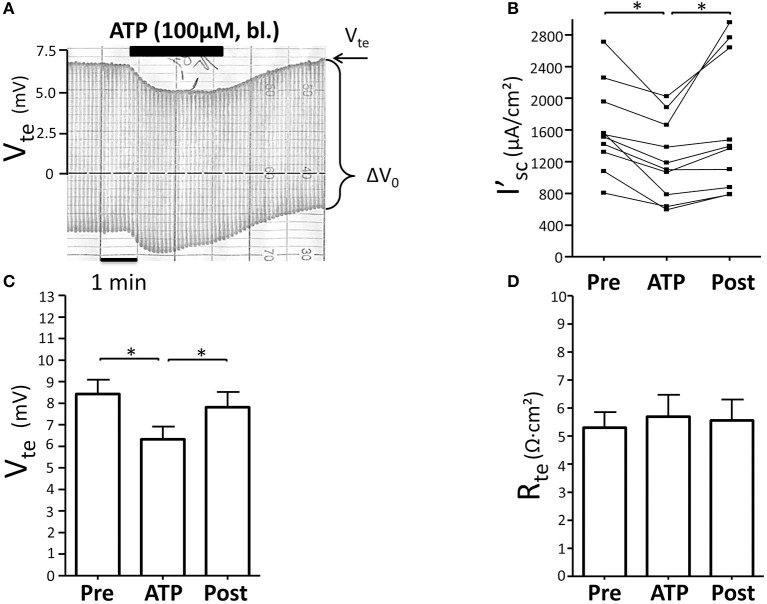
**Basolateral ATP inhibits NaCl absorption in isolated perfused mouse mTAL. (A)** Representative original trace of the basolateral ATP effect in mouse mTAL. Recording of the electrical parameters V_te_ (transepithelial voltage) and ΔV_0_ (voltage deflection) in freshly dissected and perfused mouse mTAL used to quantify transport changes. **(B)** Summary: calculated equivalent short-circuit current (I'_sc_) before, during (after 1 min) and after ATP wash-out (2 min after). (*n* = 10, ^*^p < 0.05 by student's *T*-test). **(C)** Summary: transepithelial voltage values. **(D)** Summary: transepithelial resistance values. (*n* = 10, ^*^p < 0.05 by student's *T*-test).

**Figure 2:**
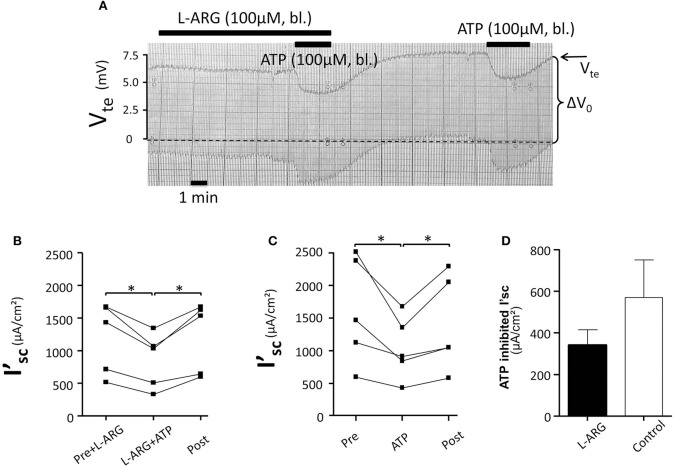
**No effect of L-arginine (100 μM) pre-incubation (7 min) (bl.) on the ATP-induced inhibition of NaCl absorption in isolated perfused mouse mTAL. (A)** Representative original trace of the basolateral ATP effect in mouse mTAL in the presence or absence of L-arginine. The order of applying ATP with or without L-arginine was alternated. Recording of the electrical parameters V_te_ (transepithelial voltage) and ΔV_0_ (voltage deflection) in freshly dissected and perfused mouse mTAL used to quantify transport changes. **(B)** Summary: calculated ATP-induced changes of the equivalent short-circuit current (I'_sc_) with pre-incubation of L-arginine (*n* = 5, ^*^p < 0.05 by student's *T*-test). **(C)** Summary: calculated ATP-induced changes of the equivalent short-circuit current (I'sc) without pre-incubation with L-arginine (*n* = 5, ^*^p < 0.05 by student's *T*-test). **(D)** Summary: ATP inhibited I'sc with and without L-arginine.

Initially we reinvestigated the effect of basolateral ATP (100 μM) in isolated perfused mouse mTALs. Figure 1A shows an original experiment, which demonstrates that addition of 100 μM ATP reversibly reduced V_te_ from ~7 to ~5 mV. The entire series shows that basolateral ATP reversibly reduced I'_sc_ and thus NaCl absorption by a mean of 384 ± 75 μA/cm^2^ (*n* = 10) (Figure 1B). No gender difference was observed in the ATP-induced I'_sc_ effect. In Figures 1C,D the respective changes in V_te_ and R_te_ are displayed and indicate that the effect of ATP on I'_sc_ is caused by a drop in V_te_, whereas R_te_ remained unchanged. These results closely recapitulate our previous results and reflect that basolateral P2X receptors trigger a significant reduction of NaCl absorption in mouse mTAL (Marques et al., 2012). In the next series of experiments, we quantified the ATP-induced transport inhibition after pre-incubation with L-arginine (100 μM) as precursor for NO formation (Figure 2). Addition or removal of L-arginine had not effect on I'_sc_ (data not shown). In paired experiments bl. ATP (100 μM) was tested with or without 7 min of pre-incubation with L-arginine. In the presence of L-arginine, bl. ATP triggered a drop of I'_sc_ by 342 ± 74 μA/cm^2^, which is not statistically significantly different from the ATP-induced transport inhibition in the absence of L-arginine 571 ± 181 μA/cm^2^ (*n* = 5). The I'_sc_ after washout of bl. ATP was higher as compared to the pre-experimental period, which we described in detail in an earlier study (Marques et al., 2012). These results show that pre-incubation with L-arginine had no effect on the bl. ATP-induced transport inhibition in mouse mTAL.

### ATP-induced transport inhibition is not affected by blocking basolateral Ca^2+^ influx

**Figure 3:**
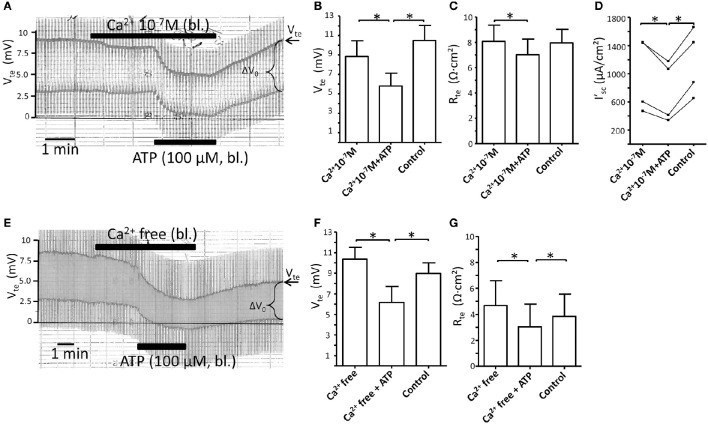
**Reduction or removal of extracellular Ca^**2+**^ does not affect the basolateral ATP-stimulated inhibition of NaCl absorption in isolated perfused mouse mTAL. (A)** Representative original trace of the basolateral ATP effect in mouse mTAL during the reduction of basolateral extracellular Ca^2+^ to 100 nM. Recording of the electrical parameters V_te_ (transepithelial voltage) and ΔV_0_ (voltage deflection) in freshly dissected and perfused mouse mTAL used to quantify transport changes. Note the slight reduction of V_te_ and ΔV_0_ after reducing extracellular Ca^2+^ from 1.3 mM to 100 nM. **(B–D)** Summary of the transepithelial voltages (V_te_), the transepithelial resistances (R_te_) and the calculated equivalent short-circuit current (I'_sc_) before, during (after 1 min) and after ATP wash-out (2 min after). (*n* = 4, ^*^p < 0.05 by student's *T*-test). **(E)** Representative original trace of the basolateral ATP effect in mouse mTAL during the removal of basolateral extracellular Ca^2+^ (5 mM EGTA). Note the continuous reduction of V_te_ and ΔV_0_ after removing extracellular Ca^2+^. **(F,G)** Summary of the V_te_ and R_te_ before, during (after 1 min) and after ATP wash-out (2 min after). (*n* = 4, ^*^p < 0.05 by student's *T*-test). Note the artificially low R_*te*_ values in **(G)**, especially during application of bl. ATP.

NO is generated by activation of Ca^2+^-dependent NOS (Alderton et al., 2001). Since P2X receptor are ligand gated, Ca^2+^ permeable cation channels, the P2X receptor stimulated increases in [Ca^2+^]_i_ can be attenuated by removal of extracellular Ca^2+^ (Jensen et al., 2007). We therefore tested the effect of blocking Ca^2+^ influx by reducing and fully removing basolateral extracellular Ca^2+^ on the ATP-induced transport inhibition. Figure 3A shows an original experiment where basolateral [Ca^2+^]_*e*_ was reduced to 100 nM. Reduced basolateral [Ca^2+^]_e_ is only tolerated by the isolated perfused tubule for a shorter period, since longer incubation (>10 min) causes a marked drop of V_te_ and R_te_ as sign for tight junction disintegration. This is seen in Figure 3A where lowering of [Ca^2+^]_e_ to 100 nM in itself caused a minor reduction of V_te_ and R_te_. After 2 min, ATP was added and induced a substantial further inhibition of transport (Δ I'_sc(ATP)_: 240 ± 53 μA/cm^2^, *n* = 4). In Figures 3B–D the summaries of the V_te_, R_te_ and I'_sc_ values are shown. In a second series of experiments, extracellular Ca^2+^ was fully removed by adding 5 mM EGTA. In Figure 3E an original trace is shown now depicting the marked effect of the removal of extracellular Ca^2+^ on V_te_ and R_te_. This effect is more drastic as compared to that shown in Figure 3A as both V_*te*_ and R_te_ continued to decrease in Ca^2+^ free solution. A longer incubation of the renal tubule (>10 min) in fully Ca^2+^ free solutions causes a complete collapse of V_te_ as sign of inter-epithelial disconnection (data not shown). Nonetheless in the time window < 10 min sufficient time prevailed to test the effect of bl. ATP (100 μM), which triggered a marked and prompt reduction of the V_*te*_. In Figures 3F,G the summaries of the V_*te*_ and R_*te*_ are shown. The injurious effect of removal of all extracelluar Ca^2+^ from the basolateral solution is clearly seen as artificially low R_te_ values (Figure 3G). This precludes reasonable calculations of I'_sc_ and therefore the size of the ATP-induced transport inhibition cannot be evaluated. However, the hallmark of the ATP effect, the prompt reduction of V_te_ is clearly seen in Figure 3E, which supports the view that the ATP effect is intact in the absolute absence of basolateral extracellular Ca^2+^. Since ATP by itself had no effect on R_te_ (Figure 1D), and since the R_te_ measurements made in the presence of ATP were acquired 2 min later in the time course following basolateral extracellular Ca^2+^ depletion, we speculate that the slight but statistically significant differences in R_te_ values in Figures 3C,G following the addition of ATP reflect the ongoing damaging effect of removing extracellular Ca^2+^. In summary, these data indicate that reducing and blocking of P2X receptor-dependent Ca^2+^ influx across the basolateral membrane has no apparent influence on the ATP-induced inhibition of NaCl absorption in mouse mTAL.

### Oxidised ATP does not affect ATP-induced increase of [Ca^2+^]_i_

**Figure 4:**
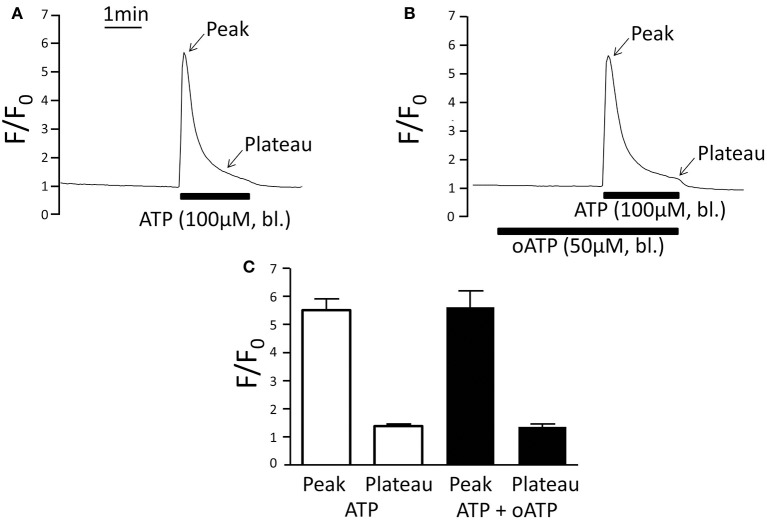
**Basolateral ATP (100 μM) stimulates very similar increases of [Ca^**2+**^]_**i**_ in the absence and presence of the P2X receptor blocker oATP. (A)** Original trace of normalized fluo-4 fluorescence (F/F_0_) without oATP (50 μM) pre-incubation. **(B)** Original trace of normalized fluo-4 fluorescence (F/F_0_) with oATP (50 μM) pre-incubation. **(C)** Summary of all results. The [Ca^2+^]_i_ plateau was measured after 2 min of ATP application.

Oxidised ATP (oATP) functions as irreversible blocker of P2X_1_, P2X_4_, and P2X_7_ receptors and in the perfused mTAL 3 min of pre-incubation with 50 μM oATP completely blocks the effect of basolateral ATP on Na^+^ and Cl^−^ absorption and the P2X receptor-stimulated intracellular alkalisation in the same tissue (Marques et al., 2012; de Bruijn et al., 2015). Using the same protocol, we studied the effect of oATP on the ATP-induced [Ca^2+^]_i_ signal. Figure 4 demonstrates that oATP did not affect the ATP-induced [Ca^2+^]_i_ peak or plateau. This is surprising because P2X receptors in general mediate Ca^2+^ influx. It was previously shown that the basolateral membrane also expresses a P2Y_2_ receptor as evidenced by absent basolateral UTP-induced [Ca^2+^]_i_ increase in the P2Y_2_ receptor knock-out mouse (Jensen et al., 2007). Therefore, the ATP-induced [Ca^2+^]_i_ increase in the presence of oATP most likely results from activation of the basolateral P2Y_2_ receptor and Ca^2+^ store release as previously documented (Jensen et al., 2007). Interestingly, oATP leads to full inhibition of the ATP-induced transport effect without having an apparent effect on the ATP-induced [Ca^2+^]_i_ response. These divergent results support that an ATP-induced [Ca^2+^]_i_ increase is not required to initiate the P2X receptor-mediated transport inhibition.

### Basolateral ATP-mediated transport inhibition is not affected by NOS inhibition

**Figure 5:**
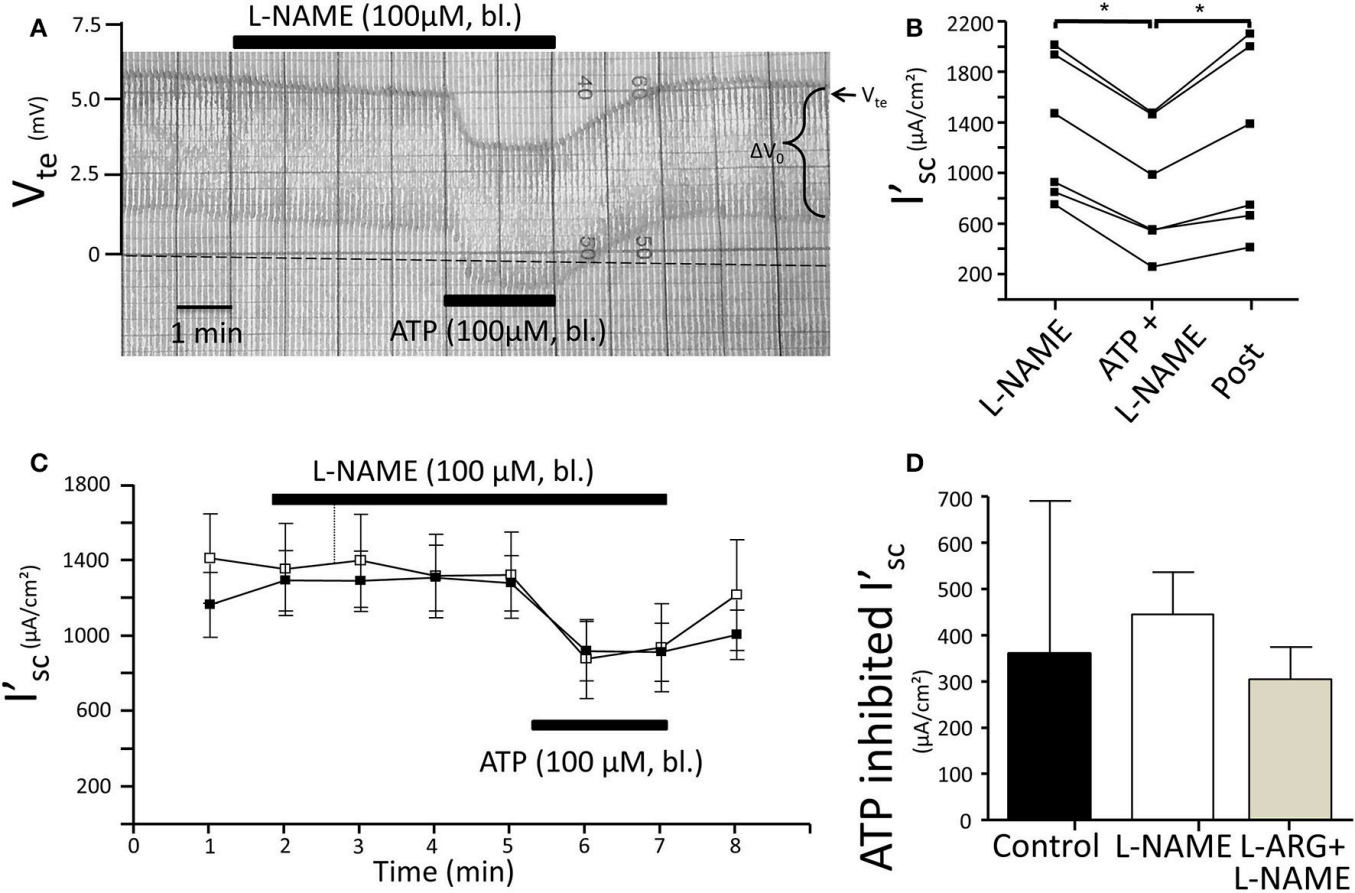
**No effect of L-NAME (100 μM) on ATP-induced inhibition of NaCl absorption in isolated perfused mouse mTAL. (A)** Representative original trace of the basolateral ATP effect in mouse mTAL during the application of basolateral L-NAME (100 μM). Recording of the electrical parameters V_te_ (transepithelial voltage) and ΔV_0_ (voltage deflection) in freshly dissected and perfused mouse mTAL used to quantify transport changes. **(B)** Summary: calculated equivalent short-circuit current (I'_sc_) of *n* = 6 experiments before, during (after 1 min) and after ATP wash-out (2 min after) in the presence of L-NAME. (*n* = 6, ^*^p < 0.05 by student's *T*-test). **(C)** Summary time curves of I'_sc_ values with basolateral ATP (100 μM) given at time point 5 min for 2 min in the absence and presence of L-NAME (*n* = 6). **(D)** Summary of ATP-inhibited I'_sc_ with and without L-NAME pre-incubation (*n* = 6) and with L-arginine/L-NAME pre-incubation (*n* = 6, ^*^p < 0.05 by student's *T*-test).

**Figure 6:**
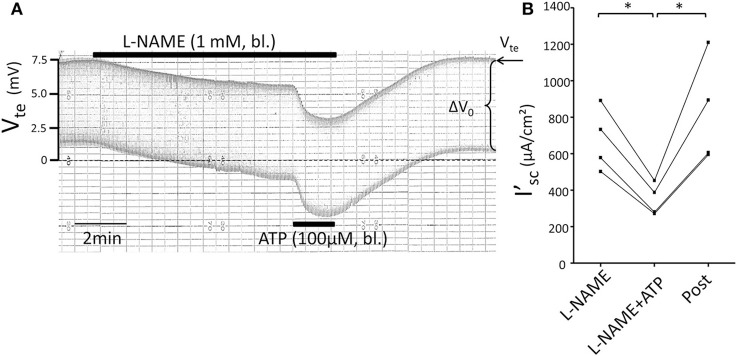
**No effect of L-NAME (1 mM) on ATP-induced inhibition of NaCl absorption in isolated perfused mouse mTAL. (A)** Representative original trace of the basolateral ATP effect in mouse mTAL during the application of basolateral L-NAME (1 mM). Recording of the electrical parameters V_te_ (transepithelial voltage) and ΔV_0_ (voltage deflection) in freshly dissected and perfused mouse mTAL used to quantify transport changes. **(B)** Summary: calculated equivalent short-circuit current (I'_sc_) before, during (after 1 min) and after ATP wash-out (2 min after) in the presence of L-NAME (*n* = 4 ^*^p < 0.05 by student's *T*-test).

**Figure 7:**
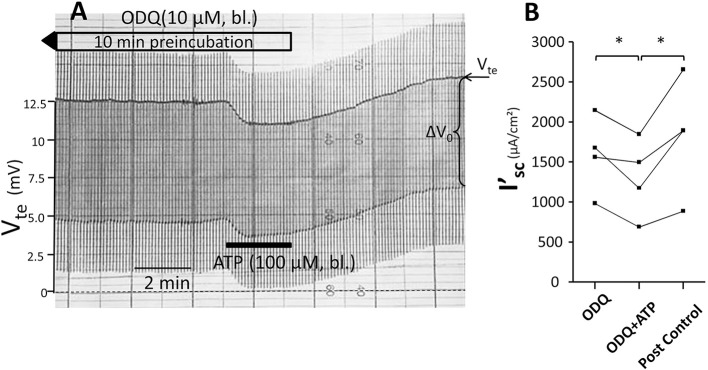
**No effect of ODQ (10 μM) on ATP-induced inhibition of NaCl absorption in isolated perfused mouse mTAL. (A)** Representative original trace of the basolateral ATP effect in mouse mTAL during the pre-incubation with 10 μM ODQ for 10 min. Recording of the electrical parameters V_te_ (transepithelial voltage) and ΔV_0_ (voltage deflection) in freshly dissected and perfused mouse mTAL used to quantify transport changes. **(B)** Summary: calculated equivalent short-circuit current (I'_sc_) before, during (after 1 min) and after ATP wash-out (2 min after) in the presence of ODQ (*n* = 4, ^*^p < 0.05 by student's *T*-test).

Subsequently, we tested the influence of L-NAME on the ATP-induced transport inhibition in isolated perfused mouse mTALs. The original experiment shown in Figure 5A indicates that basolateral addition of 100 μM L-NAME *per se* had no major effect on the transepithelial electrical transport parameters, albeit a small and very slow reduction of V_*te*_ is tentatively visible over time. After 4 min of pre-incubation with L-NAME, basolateral ATP (100 μM) was added and an acute and reversible reduction of transport can be seen. Figures 5B–D summarize the results of the entire series. These data show that irrespective of the presence of the NOS blocker L-NAME, the ATP-induced transport inhibition unaffectedly prevailed. The L-NAME (100 μM) experiments were also conducted after pre-incubation with 100 μM L-arginine and showed that bl. ATP reversibly reduced I'_sc_ by 300 ± 70 μA/cm^2^ (*n* = 5) (Figure 5D). It has been reported that L-NAME at a concentration of 3 mM blocks the ATP-induced reduction of O_2_ consumption in mTAL tubules in suspension (Silva and Garvin, 2009). We therefore repeated the above-mentioned experiments with a 10-fold higher concentration of L-NAME (1 mM) and an extended pre-incubation period of 15 min. The original experiment shown in Figure 6 indicates two things: 1. Addition of L-NAME at 1 mM led to a sustained and continuous reduction of the V_te_, which after 15 min amounted to a reduced I'_sc_ by 497 ± 98 μA/cm^2^ (*n* = 4, *p* = 0.05). In the presence of 1 mM L-NAME, the ATP reduced transport was 327 ± 44 μA/cm^2^. In summary, these results determine that L-NAME did not block the acute ATP-induced transport inhibition in the isolated perfused mouse mTAL. NO mediates its effect by activation of the soluble guanylyl cyclase, which can be irreversibly blocked by ODQ (1H-[1,2,4]oxadiazolo[4,3-a]quinoxalin-1-one) (Garthwaite et al., 1995). Therefore, we also tested the effect of ATP after pre-incubation of the mTAL with basolateral ODQ (10 μM) for 10 min. ODQ did not affect the transport parameters and the ATP-induced reduction of I'_*sc*_ was still present (Figure 7, Δ I'_sc(ATP)_: 269 ± 73 μA/cm^2^, *n* = 4). Eventually, we also tested the combination of L-NAME (1 mM) and ODQ (10 μM) to potentially block the NO signaling pathway (basolateral pre-incubation for 10 min). In the presence of both blockers, the ATP-induced effect amounted to 672 ± 111 μA/cm^2^ (*n* = 4). In summary, these experiments indicate that NO signaling is not involved in P2X receptor-mediated inhibition of Na^+^ and Cl^−^ absorption in mouse mTAL.

### The no donors SNAP and spermine nonoate do not affect the transepithelial transport in mouse mTAL

**Figure 8:**
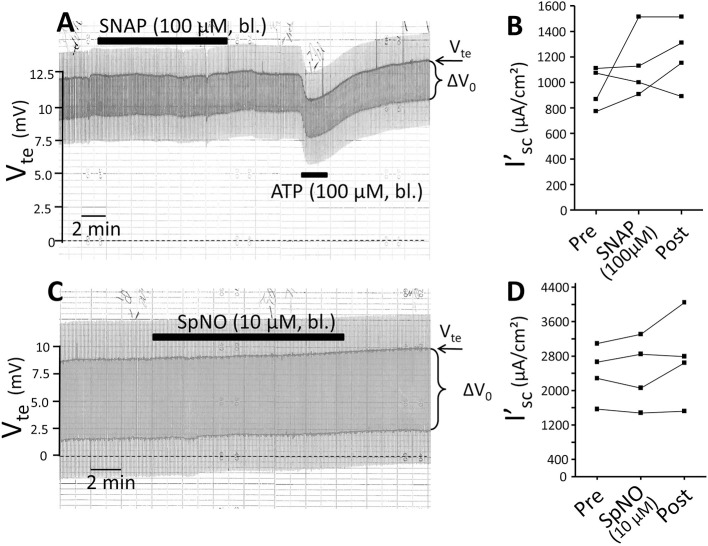
**The nitric oxide donors SNAP and Spermine NONOate have no influence on NaCl absorption in isolated perfused mouse mTAL. (A)** Representative original trace of the effect of basolateral SNAP (100 μM given for 10 min) in mouse mTAL. Recording of the electrical parameters V_te_ (transepithelial voltage) and ΔV_0_ (voltage deflection) in freshly dissected and perfused mouse mTAL used to quantify transport changes. After wash-out of SNAP basolateral ATP (100 μM) was given to test the tubule's functional viability. **(B)** Summary: calculated equivalent short-circuit current (I'_sc_) of *n* = 4 experiments before, during (after 10 min) and after SNAP wash-out (4 min after). **(C)** Representative original trace of the effect of basolateral Spermine NONOate (10 μM given for 10 min) in mouse mTAL. Recording of the electrical parameters V_te_ (transepithelial voltage) and ΔV_0_ (voltage deflection) in freshly dissected and perfused mouse mTAL used to quantify transport changes. **(D)** Summary: calculated equivalent short-circuit current (I'_sc_) of *n* = 4 experiments before, during (after 10 min) and after Spermine NONOate wash-out (4 min after).

ATP has been shown to increase NO in isolated perfused mouse mTAL as measured by DAF-2 fluorescence (Silva et al., 2006). We tested the effect of the NO donor SNAP and questioned if this could reduce transport in the mTAL. Addition of SNAP (100 μM) for 10 min did not alter transport as shown in Figure 8. After washout of SNAP, ATP was added and reversibly reduced I'_sc_. In a second series of experiments 10 μM of the NO donor SpermineNonoate (SpNO) was tested and showed no effect. In parallel experiments performed on the day SpNO was proven to lead to full vasorelaxation at 10 μM in isolated mouse basilar arteries (data not shown). This suggests that an acute elevation of NO by external application of NO donors left the resting transport characteristics unaffected in isolated perfused mouse mTAL.

## Discussion

P2X receptors are generally expressed in all types of renal epithelia, however, their functional significance has been largely elusive (Leipziger, 2016). A few observations suggest their involvement in the regulation of tubular ion transport. For example, P2X receptor agonists have been shown to modify epithelial Na^+^ channel (ENaC)-dependent whole cell currents in rat cortical collecting duct principal cells (Wildman et al., 2008) and P2X_4_ receptor activation was demonstrated to inhibit TRPM6 Mg^2+^ channel when co-expressed in HEK cells (de Baaij et al., 2014). Direct transport studies using tubular perfusion of mouse medullary thick ascending limbs (mTAL) indeed provided proof of concept that P2X receptor stimulation has substantial effects on tubular ion transport (Marques et al., 2012). Basolateral stimulation of P2X receptors inhibited about 25% of Na^+^ and Cl^−^ absorption in mTAL (Marques et al., 2012). Mouse mTALs express mRNA for the P2X_1_, P2X_4_ and P2X_5_ receptor and in knock-out mice lacking the P2X_4_ receptor subunit the effect of basolateral ATP was reduced (Marques et al., 2012). Pharmacological intervention with different unspecific P2X blockers (oATP and NF023) completely inhibited the ATP effects either on the measured short circuit current (Marques et al., 2012) or on O_2_ consumption (Silva and Garvin, 2009). P2X receptors are non-selective cation channels that also permit influx of Ca^2+^ (North, 2002). Basolateral ATP stimulated a transient followed by a sustained increase of [Ca^2+^]_*i*_ for the entire application period for ATP. This suggests sustained activation of P2X receptor-mediated influx of Ca^2+^ across the basolateral membrane, which requires extracellular Ca^2+^ (Jensen et al., 2007). The signal transduction of P2X receptors was studied and several findings suggest nitric oxide (NO) being down stream of P2X receptor activation mediating the transport inhibition. It was reported that ATP stimulates NO formation using DAF fluorescence in rat mTAL tubule suspensions (Silva et al., 2006) and it was further shown that 3 mM of L-NAME fully blocked the ATP-induced reduction of O_2_ consumption as surrogate measure of transepithelial ion transport (Silva and Garvin, 2009). This is also supported by earlier data, which showed that addition of 0.5 mM of L-arginine reversibly inhibited some 40% of Cl^−^ absorption in perfused rat cTAL where this effect was sensitive to 5 mM of the NO inhibitor L-NAME (Plato et al., 1999). The inhibitory effect of NO on the TAL was studied in further detail and the results suggested an inhibition of NKCC2 via activation of cGMP-stimulated phosphodiesterase (Ortiz and Garvin, 2001; Ortiz et al., 2001). Taken together, the published results indicate that NO negatively regulates Na^+^ and Cl^−^ absorption in rat TAL and imply that NO production distal to P2X receptor activation may explain the ATP-induced acute transport inhibition. In this study, we addressed, whether ATP-induced inhibition of Na^+^ and Cl^−^ absorption is mediated via NO signaling by directly measuring transport in the isolated perfused mouse mTAL. The generation of NO requires an increase of [Ca^2+^]_i_ to stimulate NOS (Alderton et al., 2001). We therefore removed basolateral extracellular Ca^2+^, which abolishes the [Ca^2+^]_i_ influx inflicted by basolateral addition of ATP in perfused mouse mTALs (Jensen et al., 2007). Surprisingly, P2X receptor-mediated transport inhibition was unaffected by removal of extracellular Ca^2+^. We also studied the effect of the P2X receptor blocker oATP on basolateral ATP-triggered [Ca^2+^]_i_ elevation, which is known to completely block the ATP-induced inhibition of Na^+^ and Cl^−^ absorption in murine mTAL(Marques et al., 2012). Remarkably, P2X receptor blockage with oATP had no effect on the ATP-induced increase of [Ca^2+^]_i_. The full effectiveness of oATP as blocker of ATP-induced transport inhibition has also previously been verified by measuring the effect of bl. ATP on the earlier described intracellular alkalisation (de Bruijn et al., 2015). Noteworthy, mouse mTAL also expresses a basolateral P2Y_2_ receptor which trigger intracellular Ca^2+^ store release(Jensen et al., 2007). We therefore propose that the unaffected [Ca^2+^]_i_ transient during blockage of the P2X receptor is caused by stimulation of the P2Y_2_ receptor. Interestingly, previous observations showed that oATP had no effect on the ATP-dependent [Ca^2+^]_i_ oscillations inflicted by basolateral application of α-haemolysin from *Escherichia coli* (Christensen et al., 2015), which is mainly caused by activation of the P2Y_2_ receptor. Together, these results indicate that an increase of [Ca^2+^]_i_ is not required to mediate the effect of basolateral ATP on Na^+^ and Cl^−^ absorption. This interpretation is further supported by studies, which clearly demonstrated that an increase in [Ca^2+^]_i_ does not alter the transepithelial voltage or resistance (Desfleurs et al., 1998). It was shown that stimulation of the Ca^2+^ sensing receptor (CaSR) inflicted clear transient increase in [Ca^2+^]_i_ in mTAL and cTAL from rats and in mTAL from mice without affecting the baseline or AVP-stimulated transepithelial voltage (Desfleurs et al., 1998). Similar observations were made with basolateral UTP that stimulated an increase in [Ca^2+^]_i_ in mouse mTAL via the P2Y_2_ receptor without affecting the transepithelial voltage (Jensen et al., 2007; Marques et al., 2012, 2013).

Furthermore, L-NAME even at very high concentrations did not affect the ATP-induced transport-inhibition. Moreover, the NO donor SNAP and SpNO were not able to mimic the ATP action on murine mTAL. The full bioactive potential of the NO donor SpNO to relax resistance arteries was verified in parallel experiments performed on the same day with freshly generated SpNO solutions, either applied to the vessels or the mTAL tubules. Taken together this cumulative evidence indicates that basolateral P2X receptor-mediated transport inhibition does not involve NO as second messenger.

The reason for this discrepancy is currently not understood. A role of NO as inhibitor of NaCl absorption was reported in both cortical and medullary TAL of mice and rats (Plato et al., 1999, 2000; Ortiz et al., 2001). The suggestion that ATP could inhibit NaCl absorption via NO, however, comes from rat data (Silva and Garvin, 2009) rendering the possibility of species differences. Several issues deserve consideration. As mentioned, it was previously shown that ATP is able to trigger NO production in rat mTAL tubule suspensions as measured by DAF fluorescence (Silva et al., 2006). Interestingly, this study also reported that another nucleotide, namely UTP had a similar effect on NO production. This P2 receptor agonist profile for NO production is reminiscent of the P2Y_2_ receptor, which indeed is expressed in the basolateral membrane of the mTAL (Jensen et al., 2007). Stimulation of the P2Y_2_ receptor, however, does not affect transport in this segment as documented in several studies (Marques et al., 2012, 2013). Therefore, the UTP-stimulated NO production can be taken as indirect argument for that NO does not mediate the ATP-induced transport inhibition in mTAL. The epithelial NO production triggered P2Y_2_ may have other local effects, e.g., the regulation of vascular tone in the medullary vascular bundles.

Another matter from an earlier study showed that the NOS blocker L-NAME attenuated the ATP-induced reduction of O_2_ consumption in mTAL (Silva and Garvin, 2009). O_2_ consumption can be used as an indirect measure for transepithelial transport and it was shown that extracellular application of ATP in mTAL tubule suspensions reduced the cellular O_2_ consumption with about 25%. Moreover, it was shown that the effect of ATP on O_2_ consumption was blocked by 3 mM L-NAME. We measured that higher concentrations (1 mM) of L-NAME in itself slowly reduced transepithelial transport in the perfused tubule. Thus, one will also expect L-NAME to reduce O_2_ consumption in tubule suspensions during incubation. However, this was not observed and O_2_ consumption stayed unchanged or was tentatively higher in the presence of L-NAME (Silva and Garvin, 2009). We therefore speculate that L-NAME, at higher concentration, causes cell damage and an artificial elevation of O_2_ consumption. Aside from this, there is a concern regarding the concentration of NOS inhibitors. NO-mediated vasodilation is fully blocked around 10–100 μM L-NAME (Alderton et al., 2001). The concentrations used in studies addressing the role of NO in renal tubular transport regulation have been orders of magnitude higher (Plato et al., 1999; Silva and Garvin, 2009), which obviously carries a risk for unspecific side effects. This imposes an additional uncertainty to the effectiveness of L-NAME as blocker of P2X receptor-mediated transport in mTAL.

We currently do not know the molecular mechanism of this P2X receptor-mediated inhibition of electrogenic NaCl transport in mouse mTAL. Either the trans- or the paracellular route of transport could be affected. The latter is unlikely because the ATP-induced reduction of V_*te*_ is not associated with a drop in R_te_. Thus, extracellular ATP should affect one or several of the major transport proteins, i.e., the apical NKCC2 and ROMK channel or the basolateral Na^+^-K^+^-ATPase and CLCKB chloride channel. Acute pharmacological inhibition of any these transporters can indeed have a very similar phenotypical appearance as the ATP effect describe here. P2X receptors are ligand-gated entry pathways for Na^+^ and the role of Na^+^ as a possible mediator of ATP-induced transport inhibition has so far not been addressed. Our electrophysiological approach precludes an easy access to test the role of Na^+^ in this process. Alternative approaches may, however, be feasible and should be approached in future experiments.

In summary, our studies imply a fundamental new mechanism in the regulation of renal tubular transport. Activation of basolateral ligand-gated P2X receptors markedly inhibits NaCl absorption in mouse mTAL, albeit the relevant intracellular signaling remains enigmatic. The current study does not provide support for either [Ca^2+^] or NO as important mediators of the ATP-induced transport inhibition in murine mTAL.

## Author contributions

JL designed experiments, performed some experiments, revised the figures, checked data analysis and wrote the manuscript. SS performed most experiments, analyzed the data, made the figures and corrected the manuscript. SI performed the Ca^2+^ experiments, analyzed the data, made the figure and corrected the manuscript. HP designed experiments and edited the manuscript.

### Conflict of interest statement

The authors declare that the research was conducted in the absence of any commercial or financial relationships that could be construed as a potential conflict of interest.
